# Spina Ventosa of the Thumb With Valgus Deformity in a Female With No Pulmonary Involvement: A First Report of Its Type in an Immunocompetent Adult From India

**DOI:** 10.7759/cureus.56129

**Published:** 2024-03-13

**Authors:** Sankalp Yadav, Gautam Rawal, Madhan Jeyaraman, Naveen Jeyaraman

**Affiliations:** 1 Medicine, Shri Madan Lal Khurana Chest Clinic, New Delhi, IND; 2 Respiratory Medical Critical Care, Max Super Speciality Hospital, New Delhi, IND; 3 Clinical Research, Virginia Tech India, Dr. MGR Educational and Research Institute, Chennai, IND; 4 Orthopaedics, ACS Medical College and Hospital, Dr. MGR Educational and Research Institute, Chennai, IND

**Keywords:** antituberculous treatment, tubercular dactylitis, valgus deformity, spina ventosa, mycobacterium tuberculosis (mtb)

## Abstract

Small bone involvement caused by *Mycobacterium tuberculosis* infection is an uncommon clinical entity. Usually, the condition is linked to the spread of bacteria from the lungs. It is rather uncommon for people without pulmonary seeding, trauma, or a history of tuberculosis to experience isolated episodes of spina ventosa. We describe a rare case of an immunocompetent Indian woman, aged 25, who complained of swelling in her left thumb. The diagnosis of primary tubercular dactylitis of the thumb was made after a thorough clinical and laboratory workup, and she received conservative treatment.

## Introduction

*Mycobacterium tuberculosis* causes an infection that results in tuberculosis. It poses a serious risk to public health systems and contributes significantly to both morbidity and mortality, especially in developing countries [1]. Mainly, this bacteria results in pulmonary infections, but extrapulmonary tuberculosis accounts for about 10-15% of the total tuberculosis burden [2].

Spina ventosa, also known as tubercular dactylitis, is the term used to describe tuberculosis that affects smaller bones of the hand and foot, such as the metacarpals, metatarsals, and phalanges [3]. Infection in smaller hand bones than foot bones is more common in this clinical condition [4]. Moreover, the disease is exceedingly rare after the age of five, as about 85% of cases are reported in children under the age of six [5]. In adults, it usually occurs between the ages of 20 and 50 [6]. Further, a detailed literature search revealed that no such case in an adult female has ever been reported from India, where there was no pulmonary involvement or any other foci of the bacteria beyond the thumb.

Here, we report the case of an immunocompetent female who had a swollen thumb for six months post-trauma. It was a difficult diagnosis due to the rarity of the condition; usually, these cases mimic other lesions, such as tumors. She was initiated on medical management for 12 months.

## Case presentation

A 25-year-old, married, non-diabetic Hindu woman presented with a painful swelling in her left thumb that had persisted for six months after a fall from a vehicle. She reported that the swelling was small at first, but over the past six months, it had become bigger. There was no prior history of fever, coughing, weight loss, or any other constitutional tuberculosis signs. She had never smoked and was a homemaker. Furthermore, neither she nor any of her acquaintances had a history of tuberculosis. Additionally, there was no prior record of visits to overcrowded places.

Upon general examination, the patient was found to be hemodynamically stable. There was no cyanosis, pallor, clubbing, icterus, or pretibial edema, and her systemic examination was within normal limits.

**Figure 1:**
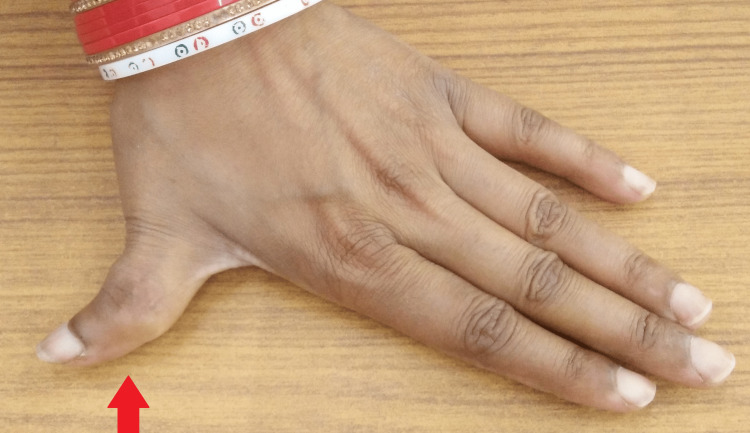
Gross image of the left thumb swelling with deformity.

**Figure 2:**
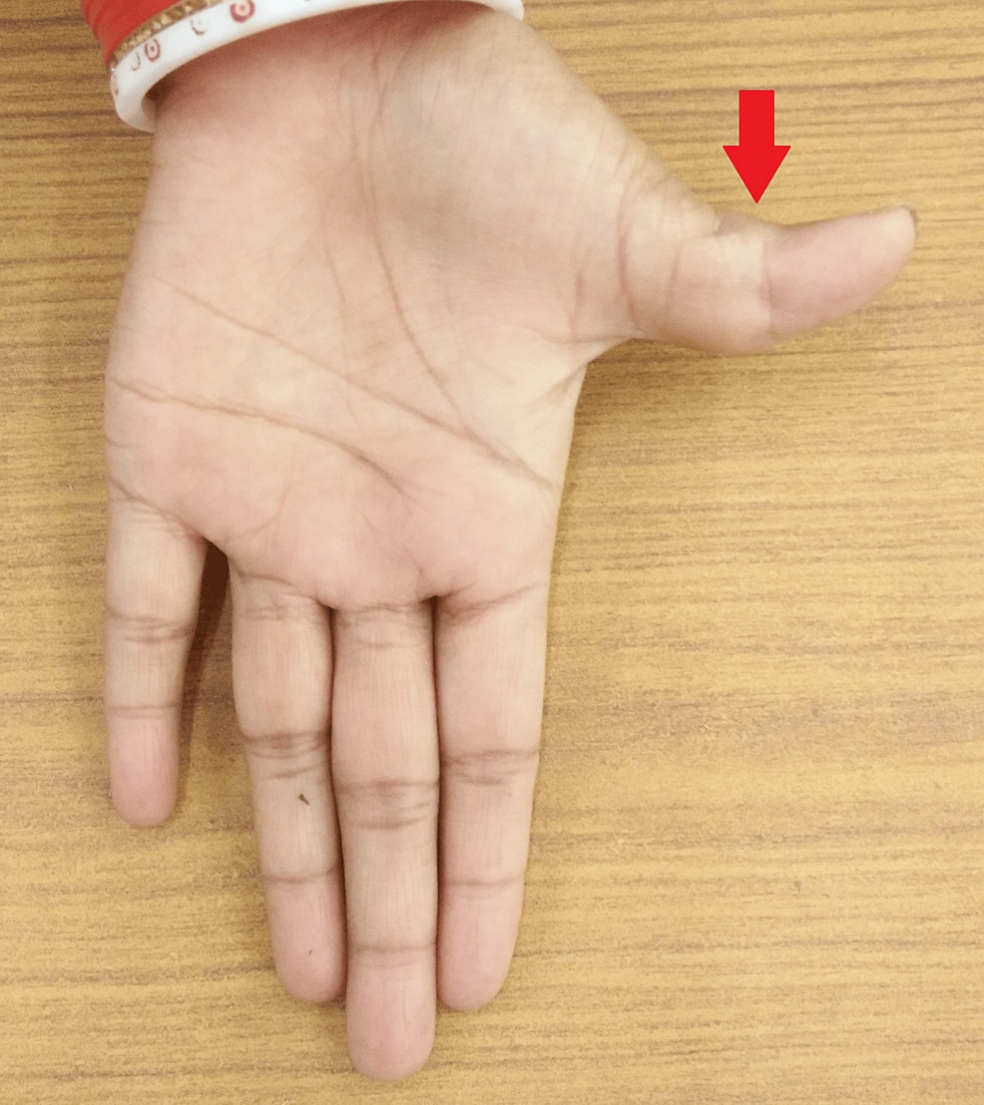
Gross image of the left thumb swelling (palmer surface).

A local examination of the left thumb revealed a visibly swollen, firm, and concentric swelling over the proximal phalanx of the thumb. There was a valgus deformity of the thumb, which was slightly tender on palpation. The range of movement was restricted at all the joints of the left thumb. However, there was no erythema or discharging sinuses (Figures 1, 2).

**Figure 3:**
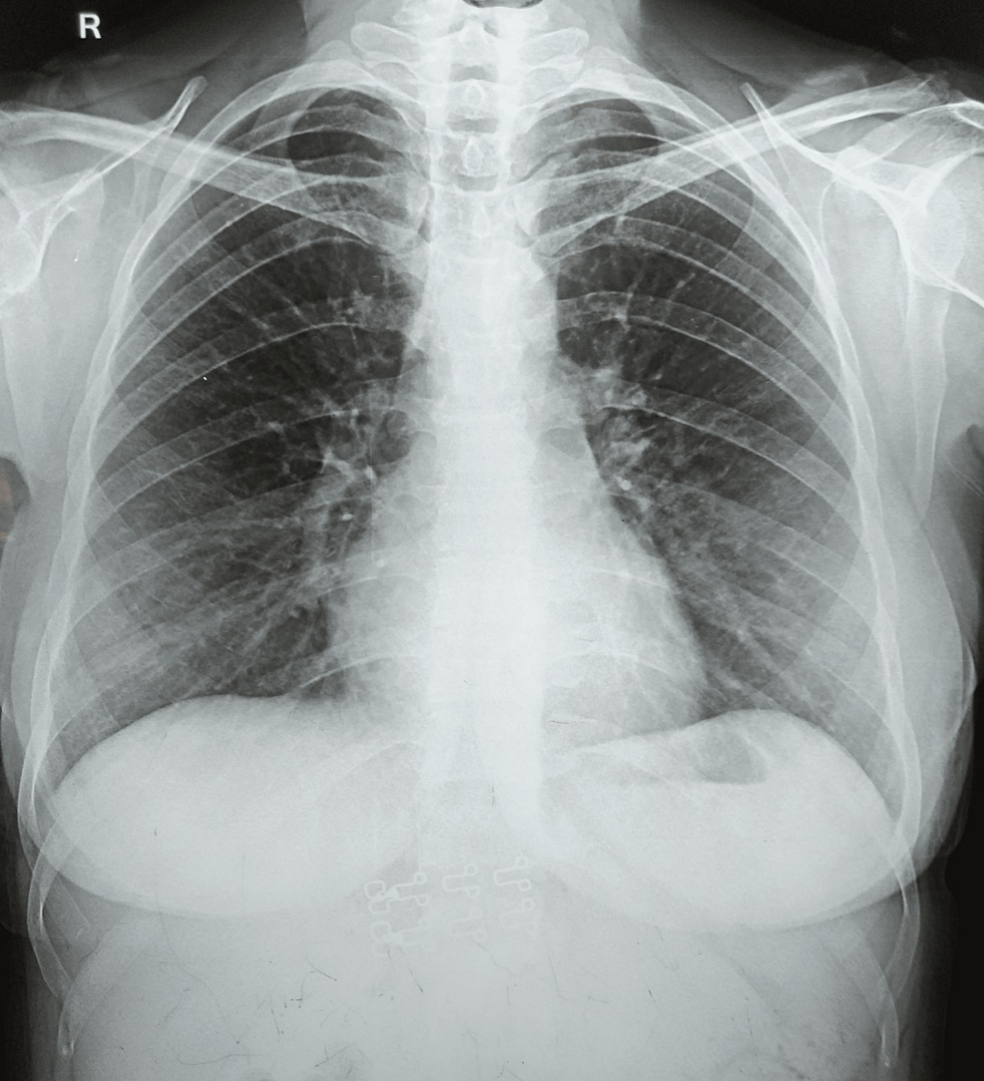
A normal chest radiograph.

Blood investigations revealed a raised erythrocyte sedimentation rate (55 mm in the first hour) and C-reactive proteins of 36 mg/L. Her hemoglobin was 10.1 g/dL, and the Mantoux test showed an induration of 21 mm. The rest of the serological tests were negative. A chest radiograph was unremarkable for any pulmonary disease (Figure 3).

**Figure 4:**
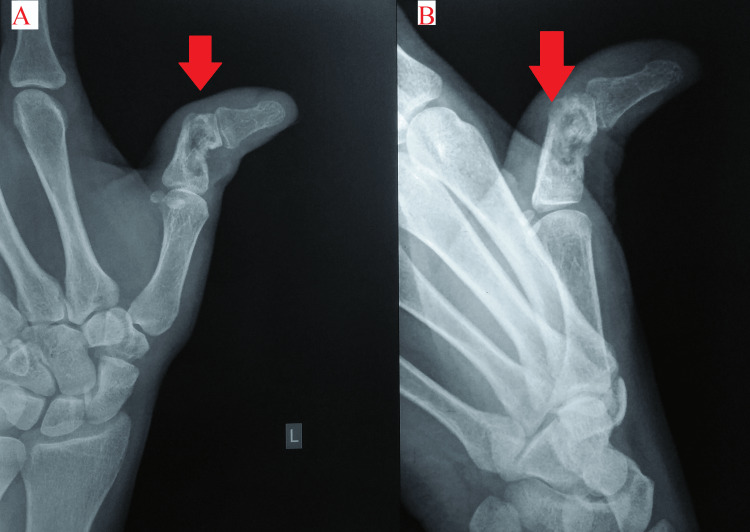
Plain radiographs of the left hand showing swollen left thumb with lesions. A: Anteroposterior view. B: Oblique view.

Plain radiographs of the left hand showed a swollen left thumb with cortical erosion in the distal part of the proximal phalanx, marked valgus deformity, and a minimal periosteal reaction (Figure 4).

**Figure 5:**
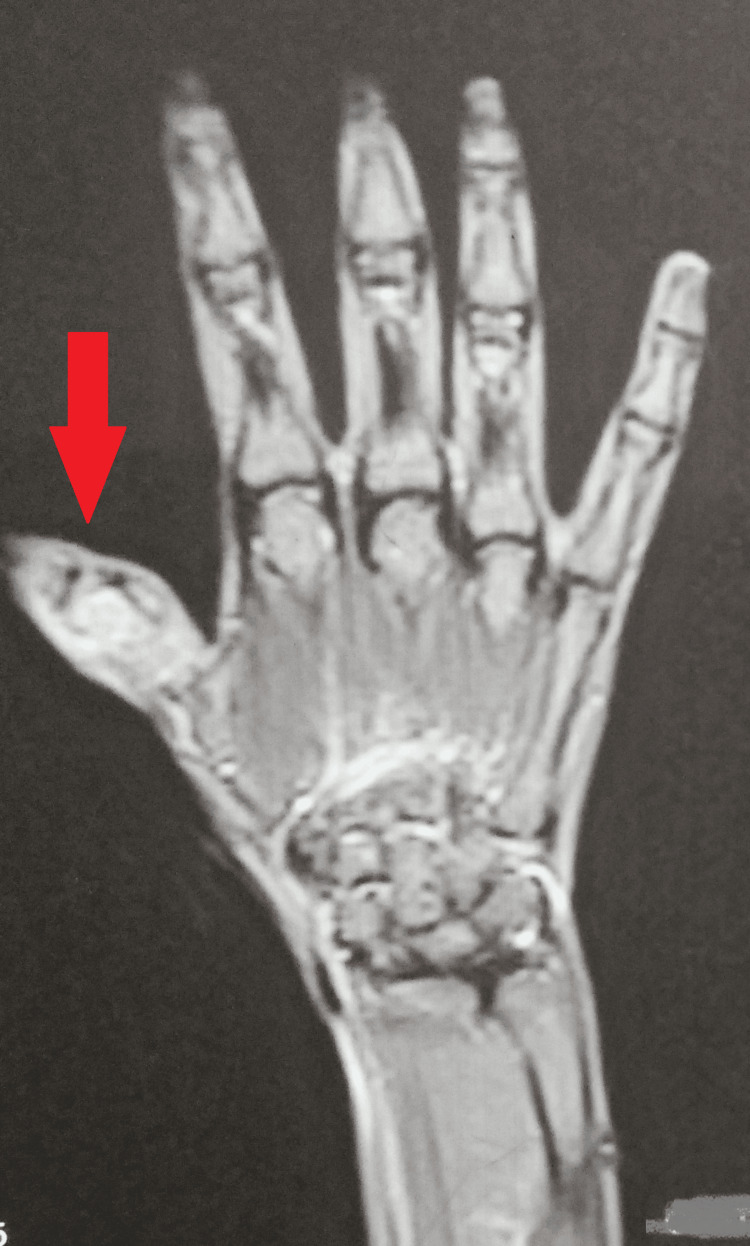
Magnetic resonance imaging of the left wrist showing significant involvement of the left thumb.

Magnetic resonance imaging of the left wrist revealed significant bone marrow edema with cortical destruction and soft tissue edema in the proximal phalanx of the left thumb, suggesting an infective etiology most likely tubercular (Figure 5).

An ultrasound-guided biopsy was performed, which indicated granulomatous inflammation with a background of necrosis and a few Langhans giant cells on histopathology. Samples sent for Gram staining, cartridge-based nucleic acid amplification testing, and culture for bacteria, fungi, and mycobacteria turned negative. However, based on the results of histopathology and radiometric investigations, a final diagnosis of spina ventosa of the left thumb was made. The patient was initiated on antituberculous chemotherapy for six months with fixed-dose combinations of rifampicin, pyrazinamide, ethambutol, and isoniazid per her weight. She was reluctant to opt for corrective surgery and continued her treatment for a further six months for a total of 12 months. After 12 months of treatment, she had a slight functional improvement with no swelling or tenderness.

## Discussion

Extrapulmonary tuberculosis is a very uncommon disease [4]. Furthermore, musculoskeletal involvement, which makes up around 10-18% of all extrapulmonary tuberculosis cases, is only observed in 1-5% of tuberculosis patients [3]. There is a gap in data on tuberculosis of the smaller bones of the hand, even in endemic nations [4].

Boyer first recognized spina ventosa, also known as tubercular dactylitis, in 1803, and Nelaton established the tuberculous etiology of this illness in 1837 [7]. It was identified by Rankin in 1886 using a histological approach, and Feilchenfeld reported roentgenographic findings in children in 1896 [4].

The proximal phalanx of the index and middle fingers is the most commonly infected area [8]. This contrasts with our patient, where it occurred in the thumb after a fall. Tuberculous dactylitis of the short bones of the hand, which was observed in our case, is known as spina ventosa due to radiographic signs of cystic growth of the small tubular bones [6].

A case similar to the present case was reported by Hassan in 2010, but the present case differs from theirs in the gender, location, and absence of lymphadenopathy [6]. Further, a detailed literature search revealed that a case similar to this case has never been reported in an adult Indian immunocompetent female where there were no pulmonary foci of the bacteria.

Based on bacteriological and histological investigations, tuberculous dactylitis can be definitively diagnosed, as noted in the present case. Sarcoidosis, gout, osteomyelitis, and malignancies are among the conditions that are included in the differential diagnosis of tuberculous dactylitis [6]. Management is essentially medical for 12 months. A situational analysis of the case leads to any further extension of treatment [4]. However, surgical debridement is recommended in cases where there is destruction of the bone and the joints [6].

## Conclusions

We present an unusual case of a young married Indian woman who complained of swelling in her left thumb. With this immunocompetent background, a high degree of suspicion was required to diagnose the patient and initiate treatment, given the absence of a history of trauma or tuberculosis. Antituberculous medications were the cornerstone of the 12-month treatment regimen.
